# Comment on Liu et al. Application of High-Flow Nasal Cannula in COVID-19: A Narrative Review. *Life* 2022, *12*, 1419

**DOI:** 10.3390/life12101625

**Published:** 2022-10-18

**Authors:** Claudia Crimi, Andrea Cortegiani

**Affiliations:** 1 Department of Clinical and Experimental Medicine, University of Catania, 95131 Catania, Italy; 2 Respiratory Medicine Unit, Policlinico “G. Rodolico-San Marco” University Hospital, Via S. Sofia, 78, 95123 Catania, Italy; 3 Department of Surgical, Oncological and Oral Science (Di.Chir.On.S.), University of Palermo, 90127 Palermo, Italy; 4 Department of Anesthesia, Intensive Care and Emergency, Policlinico Paolo Giaccone, University of Palermo, Via del Vespro, 129, 90127 Palermo, Italy

We read the article *“Application of High-Flow Nasal Cannula in COVID-19: A Narrative Review”* by Liu and colleagues [1] with great interest. The relatively recent introduction of High Flow Nasal Therapy (HFNT) into clinical practice led to growing reports on its beneficial role in the treatment of Acute Hypoxemic Respiratory Failure (AHRF) of several different origins [2], despite the presence of some conflicting evidence [3,4,5]. However, the pathophysiological abnormalities underlying hypoxemia in patients with AHRF due to viral illness caused by the pandemic might be different. This scenario should be considered as a separate clinical entity as it is, indeed, in the ERS/ATS guidelines on noninvasive ventilation [6]. Hypoxemia is common in COVID-19 patients, and is usually accompanied by an increased alveolar to arterial oxygen gradient, which may be due to intrapulmonary shunting or ventilation–perfusion mismatch. Moreover, the absence of an abnormal breathing pattern, despite the presence of hypoxemia and the presence of hypocapnia, provides a distinctive clinical presentation [7].

Liu and colleagues [1] reported an overview of the clinical application of HFNT in the management of COVID-19-related AHRF, and we appreciate their insightful summary of the available evidence. This type of noninvasive respiratory support has been widely and variably used during the pandemic, with different reported outcomes [8,9] and pending definitive conclusion [10,11,12]. Moreover, the increasing use of HFNT among patients with AHRF due to COVID-19 of different severities raises questions about the best timing of its application.

As reported by the authors, the HiFLo-COVID randomized controlled trial conducted in Colombia [10] demonstrated that HFNT significantly reduced the risk of intubation (HR 0.62 [95% CI 0.39–0.96]; *p* = 0.03) and time to clinical recovery (HR 1.39 [95% CI 1.00–1.92]; *p* = 0.047) compared to conventional oxygen therapy (COT) in patients with severe COVID-19 (PaO_2_/FiO_2_ < 200). However, in a recently published multicenter randomized controlled trial [12] performed on patients with COVID-19 pneumonia and mild hypoxemia (PaO_2_/FiO_2_ ≥ 200 and < 300), we found that the use of HFNT did not significantly reduce the likelihood of escalation of respiratory support (absolute risk difference −8.2% [95% CI −18 +1.4]; RR 0.79 [95% CI, 0.59–1.05]; *p* = 0.09) or the likelihood of clinical recovery (69.1% vs. 60.8%; absolute risk difference 8.2 [95% CI −1.5% to +18.0%], RR 1.14 [95% CI 0.98 to 1.32]) compared with COT. Thus, the intriguing pathophysiological effects of HFNT are unlikely to significantly affect the clinical course of COVID-19 pneumonia-related mild hypoxemia compared with COT. However, the study’s power was limited; therefore, a clinically meaningful benefit from HFNT in this patient population could not be definitively ruled out. Nevertheless, the results of these two trials suggest that the clinical benefit of HFNT over COT may differ according to the severity of COVID-19-related acute hypoxemic respiratory failure. In this regard, the authors should be more cautious in suggesting the use of HFNT as first-line therapy in the management of COVID-19-related hypoxemia. Physicians are often worried about oxygen levels [7] and might be encouraged to use HFNT indiscriminately as a more “effective oxygenator” due to its ability to match patients’ inspiratory peak flow, avoiding dilution with room air. Yet, HFNT imposes the use of a high amount of oxygen, especially in hypoxemic patients who require high flow settings. However, a judicious administration of oxygen and careful choice of its delivery strategy is required/mandatory, especially in the catastrophic scenario of a pandemic.

A personalized approach for the use of HFNT in COVID-19-related AHRF is crucial to improve patient outcomes, implement adequate oxygen conservation strategies and infection prevention measures, and save critical care resources. Still, uncertainty remains on the best oxygenation strategy in this patient population, and this topic deserves further research.

## References

[1] Liu C.-W., Cheng S.-L. (2022). Application of High-Flow Nasal Cannula in COVID-19: A Narrative Review. Life.

[2] Rochwerg B., Einav S., Chaudhuri D., Mancebo J., Mauri T., Helviz Y., Goligher E.C., Jaber S., Ricard J.D., Rittayamai N. (2020). The role for high flow nasal cannula as a respiratory support strategy in adults: A clinical practice guideline. Intensive Care Med..

[3] Cortegiani A., Crimi C., Sanfilippo F., Noto A., Di Falco D., Grasselli G., Gregoretti C., Giarratano A. (2019). High flow nasal therapy in immunocompromised patients with acute respiratory failure: A systematic review and meta-analysis. J. Crit. Care.

[4] Helviz Y., Einav S. (2018). A Systematic Review of the High-flow Nasal Cannula for Adult Patients. Crit. Care.

[5] Cortegiani A., Crimi C., Noto A., Helviz Y., Giarratano A., Gregoretti C., Einav S. (2019). Effect of high-flow nasal therapy on dyspnea, comfort, and respiratory rate. Crit. Care.

[6] Rochwerg B., Brochard L., Elliott M.W., Hess D., Hill N.S., Nava S., Navalesi P., Antonelli M., Brozek J., Conti G. (2017). Official ERS/ATS clinical practice guidelines: Noninvasive ventilation for acute respiratory failure. Eur. Respir. J..

[7] Tobin M.J., Laghi F., Jubran A. (2020). Why COVID-19 Silent Hypoxemia Is Baffling to Physicians. Am. J. Respir. Crit. Care Med..

[8] Crimi C., Noto A., Cortegiani A., Impellizzeri P., Elliott M., Ambrosino N., Gregoretti C. (2020). Noninvasive respiratory support in acute hypoxemic respiratory failure associated with COVID-19 and other viral infections. Minerva Anestesiol..

[9] Crimi C., Pierucci P., Renda T., Pisani L., Carlucci A. (2022). High-Flow Nasal Cannula and COVID-19: A Clinical Review. Respir. Care.

[10] Ospina-Tascon G.A., Martinez D., Gempeler A. (2022). High-Flow Oxygen vs Conventional Oxygen and Invasive Mechanical Ventilation and Clinical Recovery in Patients with Severe COVID-19-Reply. JAMA.

[11] Perkins G.D., Ji C., Connolly B.A., Couper K., Lall R., Baillie J.K., Bradley J.M., Dark P., Dave C., De Soyza A. (2022). Effect of Noninvasive Respiratory Strategies on Intubation or Mortality Among Patients with Acute Hypoxemic Respiratory Failure and COVID-19: The RECOVERY-RS Randomized Clinical Trial. JAMA.

[12] Crimi C., Noto A., Madotto F., Ippolito M., Nolasco S., Campisi R., De Vuono S., Fiorentino G., Pantazopoulos I., Chalkias A. (2022). High-flow nasal oxygen versus conventional oxygen therapy in patients with COVID-19 pneumonia and mild hypoxaemia: A randomised controlled trial. Thorax.

